# Effect of external magnetic field and doping on electronic and thermodynamic properties of planer and buckled silicene monolayer

**DOI:** 10.1038/s41598-022-26353-1

**Published:** 2022-12-24

**Authors:** Mona Abdi, Erfan Norian, Bander Astinchap

**Affiliations:** 1 Department of Physics, Faculty of Science, University of Kurdistan, Sanandaj, Kurdistan 66177-15175 Iran; 2 Research Center for Nanotechnology, University of Kurdistan, Sanandaj, Kurdistan 66177-15175 Iran

**Keywords:** Materials science, Nanoscience and technology

## Abstract

In this research, the electronic and thermodynamic properties of the planer and buckled silicene monolayer under an external magnetic field and doping using the tight-binding (TB) model and the Green function approach are investigated. Also, the dependence of the electronic heat capacity and magnetic susceptibility with temperature, external magnetic field, electron, and hole doping for the planer and buckled silicene monolayer is calculated. Our numerical calculation exhibits that the planer and buckled silicene monolayer have a zero band gap. We find that the electronic heat capacity increases (decreases) by applying an external magnetic field, and electron and hole doping at lower (higher) temperatures due to the increase in the thermal energy (scattering and collision) of the charge carriers. Finally, we observe that the planer and buckled silicene monolayer is antiferromagnetic, which is changed to the ferromagnetic phase when an external magnetic field and doping are applied, which makes the silicene monolayer suitable for spintronic applications.

## Introduction

In 2004^1^, Graphene was discovered as the first two-dimensional structure. Graphene, which combines carbon atoms to have a honeycomb structure, attracts a lot of attention from theoretical and experimental researchers because of its electrical, and conductivity properties^2–5^. The unique properties of graphene led to studied of other elements of group IV such as silicene and germanium with a similar structure to graphene^6–11^. Silicene in 1994 was predicted by theoretical researchers and then scanning tunneling microscopic (STM) images showed silicene nanoribbons and sheets on a silver crystal. Silicene with a hexagonal structure is similar to graphene which has different crystalline forms due to its hybridization^11, 12^. According to hybridization, silicene is classified into two groups: planar silicene with hybridization sp^2^ and buckled silicene with hybridization between sp^2^ and sp^3^^10^. Many theoretical researchers used the TB method and density functional theory (DFT) to investigate the optical, electrical, and thermal properties of silicene sheets^13–16^. Guzman-Verri and Voon used TB to study the electrical properties of silicene sheets and silicene nanotubes. Their results showed that silicene sheets have a metallic or zero band gap^7^. Drummond et al. calculated the electronic structure of silicene in the presence of an external electric field oriented perpendicular to the monolayer of Si atoms. They showed that the electric field causes a tunable band gap at the Dirac point^17^. Feyzi and Chegel investigated the electronic heat capacity, electrical, and thermal conductivity of planar and buckled silicene monolayer through TB approximation by applying doping atoms^18^. Chegel et al. surveyed the density of states and electrical and optical conductivities of monolayer and bilayer silicene using TB approximation and Green function method by applying the electric field on doped bilayer silicene^19^. Yarmohammadi studied electronic heat capacity and magnetic susceptibility of ferromagnetic silicene sheets under strain that is focused on the effects of an external static electric field in the presence of strain on electronic heat capacity and magnetic susceptibility of a ferromagnetic silicene sheet^20^. In this paper, we study the effect of the external magnetic field, electron, and hole doping on density of states, electronic heat capacity, and magnetic susceptibility of planar and buckled silicene monolayer in TB approximation. We applied the Green function for calculating the electronic heat capacity and magnetic susceptibility. We investigate dependence of electronic heat capacity and magnetic susceptibility with temperature under the effect of external magnetic field, electron, and hole doping. The setup of the remains of the paper is as follows. In “Theory and model” section, we describe the TB model and Green’s function for computing the heat capacity and magnetic susceptibility properties. We discuss the numerical results related to the effects of external magnetic field and electron and hole doping on thermodynamic properties in “Result and discussion” section and we pull the original results in “Conclusions”.

## Theory and model

**Figure 1:**
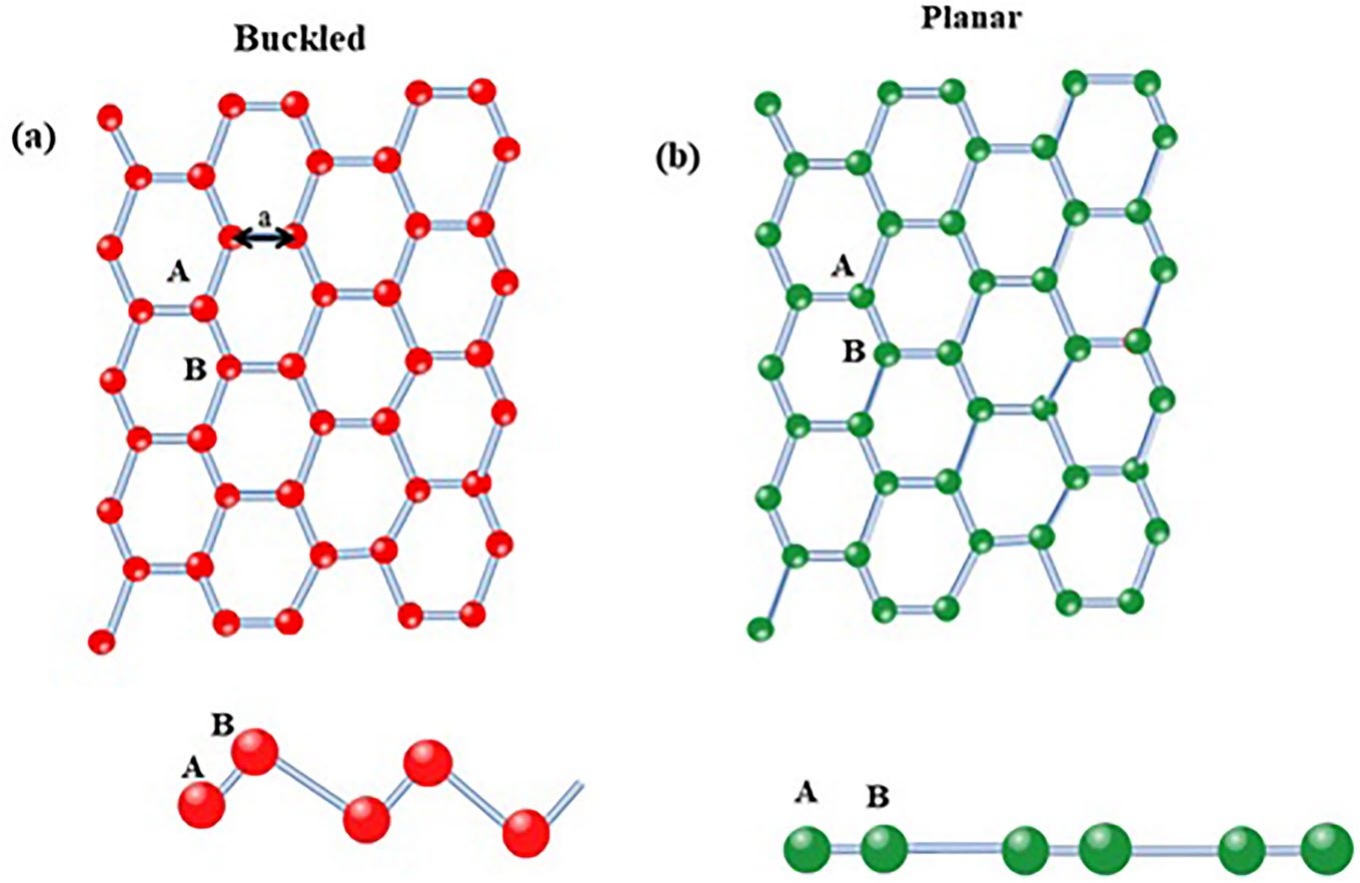
A schematic of the planar and buckled silicene monolayer.

In this section, we calculated the TB Hamiltonian of the planar and buckled silicene monolayer in the presence of an external magnetic field, electron, and hole doping. Then we investigate the electronic and thermodynamic properties of the planar and buckled silicene monolayer. Figure 1 shows a schematic of the planar and buckled silicene monolayer.

In the first, we calculate TB Hamiltonian including the first nearest neighbors, to all 3s and 3p orbitals atom Silicene. According to Fig. 1, the unit cell consists of A and B atoms and the three nearest neighbor vectors defined below:

1 $${\Delta }_{1}=\left(\frac{a}{\sqrt{3}} ,0,\frac{acot\varphi }{\sqrt{3}}\right).{\Delta }_{2}=\left(-\frac{a}{2\sqrt{3}} ,\frac{a}{2},\frac{acot\varphi }{\sqrt{3}}\right). {\Delta }_{3}=\left(-\frac{a}{2\sqrt{3}} ,-\frac{a}{2},\frac{acot\varphi }{\sqrt{3}}\right)$$

Here, the planar and buckled silicene are φ = 90° and φ = 101.7°, respectively, and the lattice constant defines planar and buckled silicene a = 3.86 Å and a = 3.89 Å^21^. Given that the unit cell consists of A and B atoms and each sub-lattice consists of four orbitals (s, p_x_, p_y_, p_z_). Therefore Hamiltonian in terms of the operator's creation and annihilation is written as follows:

2 $${H}_{t.b}^{\sigma }=\sum_{i,j,\sigma ,s,p,\alpha ,\beta }{\epsilon }_{i,j}^{\sigma ,\alpha ,\beta }{\delta }_{i,j}{\delta }_{\alpha \beta }{\delta }_{s,p}{a}_{i,s}^{\dagger,\sigma ,\alpha }{a}_{j,p}^{\sigma ,\beta }+\sum_{i,j,\sigma ,\alpha ,\beta ,s,p,\Delta }{t}_{i,s,j,p}^{\alpha ,\beta }(\Delta ){a}_{i,s}^{\dagger,\sigma ,\alpha }{a}_{j,p}^{\sigma ,\beta }-\sum_{i,j,\sigma ,\alpha ,\beta ,s,p}(g{\mu }_{B}\mathrm{B\sigma }+\upmu ) {\delta }_{i,j}{\delta }_{\alpha \beta }{\delta }_{s,p}{a}_{i,s}^{\dagger,\sigma ,\alpha }{a}_{j,p}^{\sigma ,\beta }.$$

where $\alpha  and \beta$ determine sub-lattice, s and p denote the orbitals for each atom(s,${p}_{x},{p}_{y},{p}_{z}$),${\epsilon }_{i,j}^{\sigma ,\alpha ,\beta }$ and ${t}_{i,s,j,p}^{\alpha ,\beta }\left(\Delta \right)$ onsite energy of silicene atoms and the hopping energy between different neighboring sites, respectively. Operator ${a}_{i,s}^{\dagger,\sigma ,\alpha }({a}_{i,s}^{\sigma ,\beta })$ is the creation (annihilating) of an electron in sub-lattice $\alpha , and  \beta$ in orbitals s with unit cell i. $\Delta$ refers to unit cell vectors of the nearest neighbors.${\mu }_{B}$ and g is the Bohr magnetron constant and gyromagnetic constant, respectively. The TB model is determined by the hopping integrals between the different orbitals, described, in the framework of a Slater-Koster explained, in terms of σ and π^22^. In Table 1 the hopping parameter is shown^7^.

**Table 1:** The hopping parameters for silicene in the unit (eV)^7^.

${E}_{s}$	${E}_{p}$	${t}_{ss\sigma }^{AB}$	${t}_{sp\sigma }^{AB}$	${t}_{pp\sigma }^{AB}$	${t}_{pp\pi }^{AB}$
− 4.04497	1.0297	− 2.066	2.0850	3.1837	0.9488

To rewrite the Hamiltonian Eq. (2) in the reciprocal space, we define the ${\upxi }_{\mathrm{k}}^{\dagger\upsigma }=({\mathrm{s}}_{\mathrm{k},\mathrm{A}}^{\dagger\upsigma },{\mathrm{p}}_{\mathrm{x},\mathrm{k},\mathrm{A}}^{\dagger\upsigma },{\mathrm{p}}_{\mathrm{y},\mathrm{k},\mathrm{A}}^{\dagger\upsigma },{\mathrm{p}}_{\mathrm{z},\mathrm{k},\mathrm{A}}^{\dagger\upsigma },{\mathrm{s}}_{\mathrm{k},\mathrm{B}}^{\dagger\upsigma }{,\mathrm{p}}_{\mathrm{x},\mathrm{k},\mathrm{B}}^{\dagger\upsigma }{,\mathrm{p}}_{\mathrm{y},\mathrm{k},\mathrm{B},}^{\dagger\upsigma },{\mathrm{p}}_{\mathrm{z},\mathrm{k},\mathrm{B},}^{\dagger\upsigma })$ creation vector. ${\upxi }_{\overrightarrow{\mathrm{k}}}^{\upsigma \dagger}$ leads to ${\mathrm{H}}_{\mathrm{t}.\mathrm{b}}^{\upsigma }=\sum_{\overrightarrow{\mathrm{k}},\upsigma }{\upxi }_{\overrightarrow{k}}^{\upsigma \dagger}{\mathrm{H}}_{\overrightarrow{\mathrm{k}}}^{\upsigma }{\upxi }_{\overrightarrow{\mathrm{k}}}^{\upsigma }$, which the Hamiltonian is calculated in the reciprocal space as follows:

3 $${H}_{t.b}^{\sigma }=\left[\begin{array}{ll}{H}_{AA}^{\sigma }-(g{\mu }_{B}\mathrm{B\sigma }+\upmu )& {H}_{AB}^{\sigma }\\ {H}_{BA}^{\sigma }& {H}_{BB}^{\sigma }-(g{\mu }_{B}\mathrm{B\sigma }+\upmu )\end{array}\right]$$

That $${H}_{AA}^{\sigma }=\left[\begin{array}{c}\begin{array}{ccc}{H}_{{s}_{A},{s}_{A}}^{\sigma }& {H}_{{s}_{A},{p}_{x,A}}^{\sigma }& {H}_{{s}_{A},{p}_{y,A}}^{\sigma }\\ {H}_{{p}_{x,A},{s}_{A}}^{\sigma }& {H}_{{p}_{x,A},{p}_{x,A}}^{\sigma }& {H}_{{p}_{x,A},{p}_{y,A}}^{\sigma }\\ {H}_{{p}_{y,A},{s}_{A}}^{\sigma }& {H}_{{p}_{y,A},{p}_{x,A}}^{\sigma }& {H}_{{p}_{y,A},{p}_{y,A}}^{\sigma }\end{array}\begin{array}{c}{H}_{{s}_{A},{p}_{z,A}}^{\sigma }\\ {H}_{{p}_{x,A},{p}_{z,A}}^{\sigma }\\ {H}_{{p}_{y,A},{p}_{z,A}}^{\sigma }\end{array}\\ \begin{array}{cc}{H}_{{p}_{z,A},{s}_{A}}^{\sigma }& {H}_{{p}_{z,A},{p}_{x,A}}^{\sigma }\end{array}\begin{array}{cc}{H}_{{p}_{z,A},{p}_{y,A}}^{\sigma }& {H}_{{p}_{z,A},{p}_{z,A}}^{\sigma }\end{array}\end{array}\right]$$ and $${H}_{AB}^{\sigma }=\left[\begin{array}{c}\begin{array}{ccc}{H}_{{s}_{A},{s}_{B}}^{\sigma }& {H}_{{s}_{A},{p}_{x,B}}^{\sigma }& {H}_{{s}_{A},{p}_{y,B}}^{\sigma }\\ {H}_{{p}_{x,A},{s}_{B}}^{\sigma }& {H}_{{p}_{x,A},{p}_{x,B}}^{\sigma }& {H}_{{p}_{x,A},{p}_{y,B}}^{\sigma }\\ {H}_{{p}_{y,A},{s}_{B}}^{\sigma }& {H}_{{p}_{y,A},{p}_{x,B}}^{\sigma }& {H}_{{p}_{y,A},{p}_{y,B}}^{\sigma }\end{array}\begin{array}{c}{H}_{{s}_{A},{p}_{z,B}}^{\sigma }\\ {H}_{{p}_{x,A},{p}_{z,B}}^{\sigma }\\ {H}_{{p}_{y,A},{p}_{z,B}}^{\sigma }\end{array}\\ \begin{array}{cc}{H}_{{p}_{z,A},{s}_{B}}^{\sigma }& {H}_{{p}_{z,A},{p}_{x,B}}^{\sigma }\end{array}\begin{array}{cc}{H}_{{p}_{z,A},{p}_{y,B}}^{\sigma }& {H}_{{p}_{z,A},{p}_{z,B}}^{\sigma }\end{array}\end{array}\right]$$

We calculated the Hamiltonian matrix Eq. (3) in detail in appendix A. Using the energy spectrum obtained from the TB model Hamiltonian matrices, we can write the Hamiltonian by:

4 $$H=\sum_{k,\sigma ,\eta }{E}_{\eta }^{\sigma }{c}_{\eta ,k}^{\dagger,\sigma }{c}_{\eta ,k}^{\sigma }.$$

According to Eq. (4), the electron Green function is given by:

5 $${\mathrm{G}}_{\upeta }^{\upsigma }\left(\mathrm{k},\uptau \right)=-\langle {\mathrm{T}}_{\uptau }\left({\mathrm{C}}_{\upeta }^{\upsigma }\left(\mathrm{k},\uptau \right){\mathrm{C}}_{\upeta }^{\upsigma ,\dagger}\left(\mathrm{k},0\right)\right)\rangle ,$$

here, τ is imaginary time, and the Fourier transformations of the electronic Green's function are obtained as follows:

6 $${\mathrm{G}}_{\upeta }^{\upsigma }\left(\mathrm{k},\mathrm{ i}{\upomega }_{\mathrm{n}}\right)={\int }_{0}^{\frac{1}{{\mathrm{k}}_{\mathrm{B}}\mathrm{T}}}{\mathrm{e}}^{\mathrm{i\omega }{\uptau }_{\mathrm{n}}}{\mathrm{G}}_{\upeta }^{\upsigma }\left(\mathrm{k},\uptau \right)=\frac{1}{\mathrm{i}{\upomega }_{\mathrm{n}}-{\mathrm{E}}_{\mathrm{\alpha }}^{\upsigma }}.$$

where T and ${\upomega }_{\mathrm{n}}=(2n+1)\pi {k}_{B}T$ are equilibrium temperature and fermions Matsubara frequency, respectively. We can define the density of states of planar and buckle silicene monolayer as follows:

7 $$\mathrm{g}\left(\upvarepsilon \right)=-\frac{1}{2\mathrm{\pi N}}\sum_{\mathrm{k},\mathrm{n},\upsigma ,\upeta }\mathrm{Im}\left({\mathrm{G}}_{\upeta }^{\upsigma }\left(\mathrm{k},\mathrm{i}{\upomega }_{\mathrm{n}}\to\upvarepsilon +\mathrm{i}{0}^{+}\right)\right).$$

Here, N is the number of atoms in the unit cell. In the following, we investigate the electronic heat capacity and magnetic susceptibility for the planar and buckled silicene monolayer with the existence of the external magnetic field, hole, and electron doping. The electronic heat capacity and magnetic susceptibility are defined in terms of the density of states^23, 24^. The electronic heat capacity and magnetic susceptibility are given as a function of the Green function as follows:

8 $$\chi \left(T\right)={\int }_{-\infty }^{+\infty }d\varepsilon (-\frac{\partial {n}_{f}\left(\varepsilon ,T\right)}{\partial \varepsilon })g\left(\varepsilon \right)=-\frac{1}{2\pi N{k}_{B}T}\sum_{k,\sigma ,\eta }{\int }_{-\infty }^{+\infty }d\varepsilon \frac{{e}^{\frac{\varepsilon }{{k}_{B}T}}}{{\left(1+{e}^{\frac{\varepsilon }{{k}_{B}T}}\right)}^{2}} Im\left({G}_{\eta }^{\sigma }\left(k,\varepsilon \right)\right).$$

And

$$C\left(T\right)={\int }_{-\infty }^{+\infty }d\varepsilon (\frac{\partial {n}_{f}\left(\varepsilon ,T\right)}{\partial T})g\left(\varepsilon \right)=-\frac{1}{2\pi N{k}_{B}T}\sum_{k,\sigma ,\eta }{\int }_{-\infty }^{+\infty }d\varepsilon \frac{{\varepsilon }^{2}{e}^{\frac{\varepsilon }{{k}_{B}T}}}{{\left(1+{e}^{\frac{\varepsilon }{{k}_{B}T}}\right)}^{2}} Im\left({G}_{\eta }^{\sigma }\left(k,\varepsilon \right)\right).$$

$g(\varepsilon )$ is the density of states in the relation (7), ${n}_{f}\left(\varepsilon ,T\right)=\frac{1}{1+{e}^{\beta \varepsilon }}$ the function is the Dirac-Fermi distribution.

## Result and discussion

**Figure 2:**
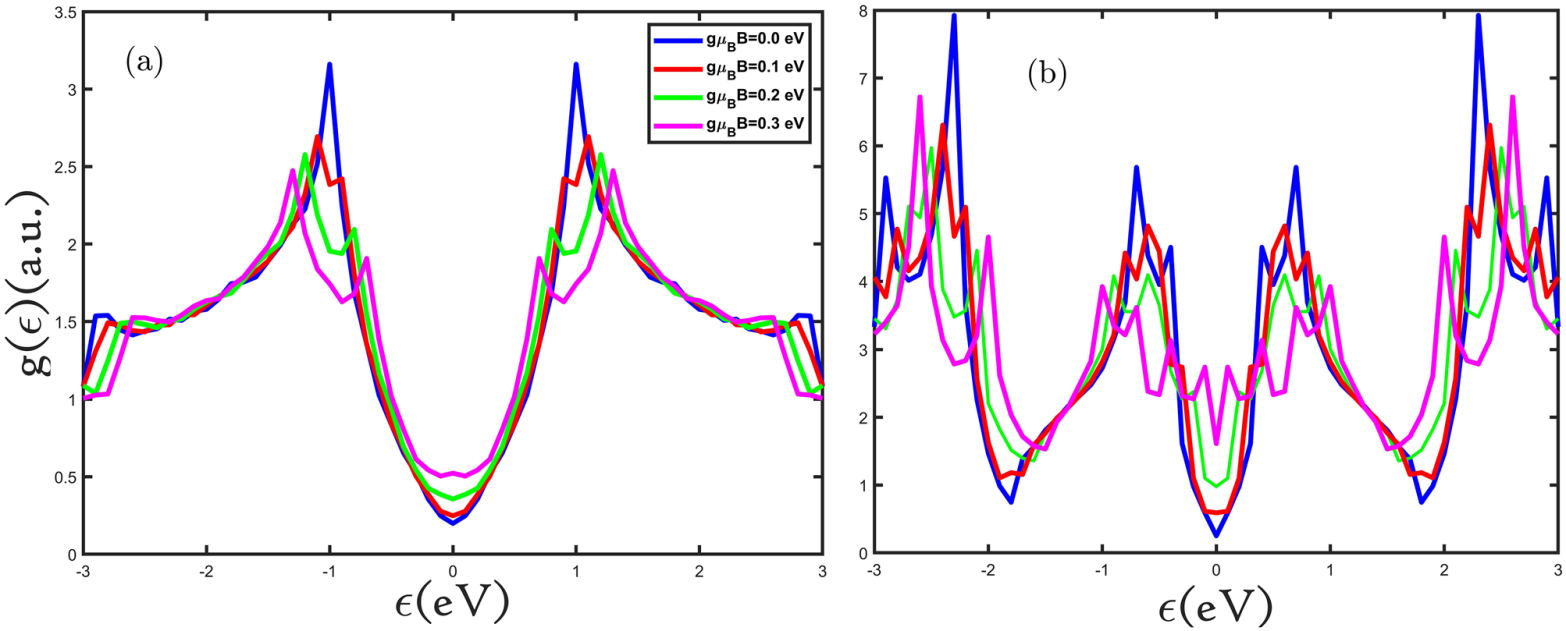
(**a**,**b**) The electronic density of states of the planar and buckled silicene monolayer in the presence of the external magnetic field.

In the following, we study the results of the density of states, electronic heat capacity, and magnetic susceptibility features of the planar and buckled silicene monolayer with the existence of the applied external magnetic field, electron, and hole doping. We have used the TB model Hamiltonian to explain the electronic and thermodynamics of the planar and buckled silicene monolayer. The density of states of planar and buckled silicene monolayer for different values of the external magnetic field is shown in Fig. 2a,b. We can see that in the absence of the external magnetic field of the planar and buckled silicene monolayer band gap is nearly zero and has metallic properties^25^. By applying an external magnetic field perpendicular to the planar and buckled silicene monolayer, its metallic properties increased. From Fig. 2a,b, we can adjust the band gap, by applying a magnetic field. In crystal solids due to the presence of saddle points of energy dispersion in momentum space the van Hove singularity in the density of states exists, which is a kink in the density of states. An interesting feature we see in Fig. 2a,b is that in the presence of the external magnetic field each Van Hove singularity is split into two singularities and with increasing the external magnetic field, the distance between splits increases. According to Zeeman Effect, the external magnetic field leads to breaking the degeneracy of the energy levels in the valence (conduction) bands at the K point. In other words, the Fermi energy approaches the van Hove energy by applying an external magnetic field, and the VHS peak in DOS splits into two new ones. Our results are consistent with the works done on the MoS_2_ monolayer and SiC bilayer^26, 27^.

**Figure 3:**
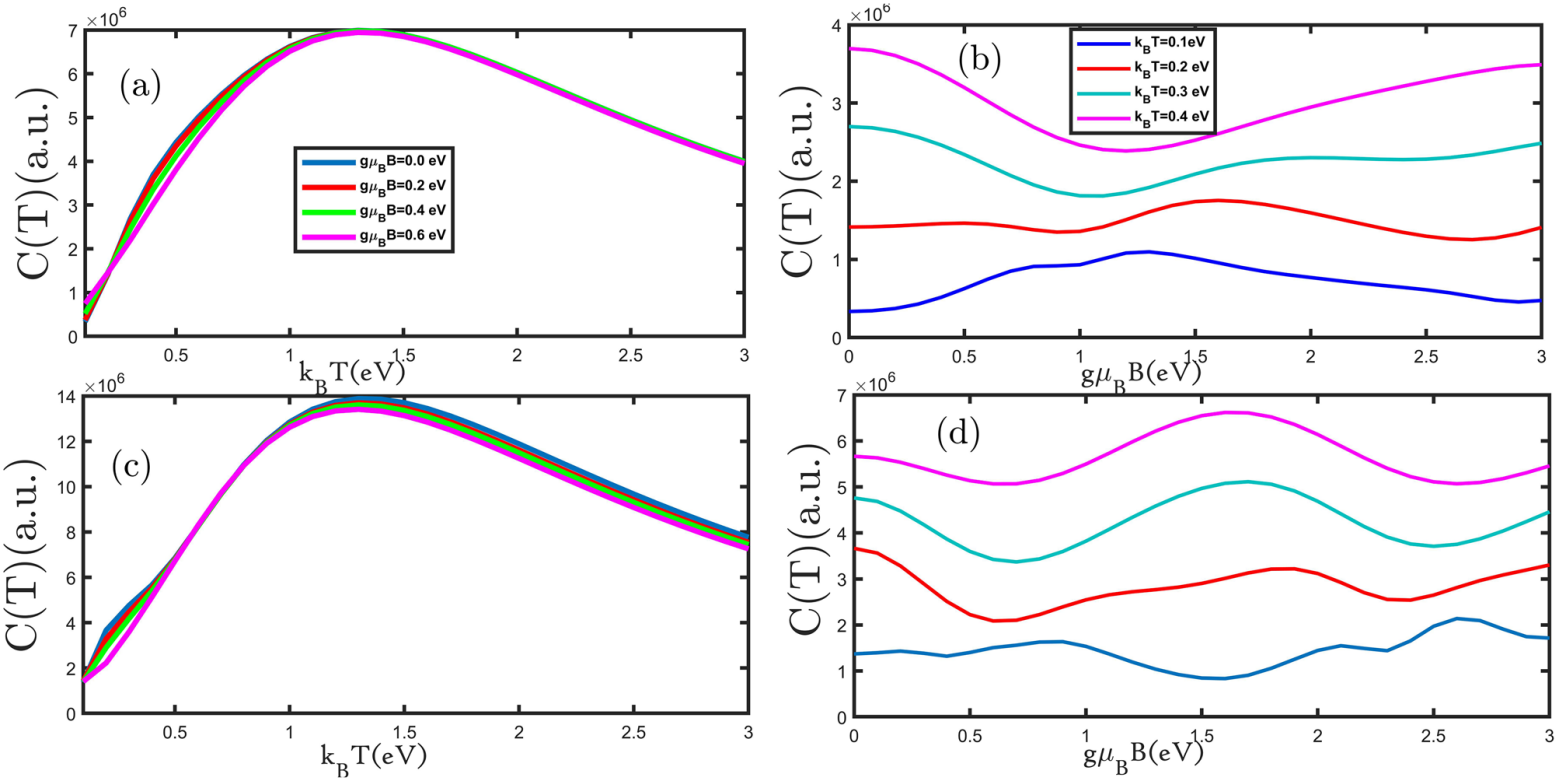
(**a**–**d**) The electronic heat capacity as a function of the temperature (external magnetic field) for planar and buckled silicene monolayers in the presence of different external magnetic fields (temperatures), respectively.

In Fig. 3a,c, we present the electronic heat capacity for planar and buckled silicene monolayers as a function of the temperature for different values of the external magnetic field. As can be seen, the electronic heat capacity in all curves increases with increasing temperature until reaching Schottky anomaly^24^, which appears at temperatures range of 1.0 eV < k_B_T < 1.5 eV. The electronic heat capacity for the temperatures of k_B_T $>1.5 \; \text{eV}$ decreases with increasing an external magnetic field in Fig. 3a,c. In the low-temperature region, the curves increase with an increase in temperature until the Schottky anomaly peak because, with increasing temperature, more electrons can move from the valence band to the conduction band, which causes increased curves. Due to the increase in the rate of electrons scattering, the curves decrease when the temperature rises. At temperatures close to zero, only low energy levels are occupied, and here the probability of electron transfer to higher levels is much lower. So, applying the magnetic field (hole and electron doping) provides the energy to transfer electrons. Therefore, at the first by applying the magnetic field (hole and electron doping) the curves increase at a fixed temperature. In Fig. 3a,c, we can see that the maximum electronic heat capacity decreased with an increasing magnetic field. Based on this, we show that the majority of carriers are electrons and increase with an increasing magnetic field^28^. Karimi et al. have achieved similar results by applying an external magnetic field for bilayer graphene^29^. The electronic heat capacity at a small range of temperatures increased with the increasing external magnetic field because the band gap decreased applying an increasing external magnetic field to according Einstein's model $\mathrm{C}(\mathrm{T})\approx {\mathrm{e}}^{-{\upvarepsilon }_{\mathrm{g}}/{\mathrm{k}}_{\mathrm{B}}\mathrm{T}}$^24^. The electronic heat capacity is increased because the band gap is decreasing as seen in Fig. 2a,b. To better examine changes in the electronic heat capacity with the external magnetic field, we plot the electronic heat capacity in terms of the external magnetic field for the planar and buckled silicene monolayer in Fig. 3b,d. In Fig. 3b, the electronic heat capacity of the planar silicene monolayer as a function of the external magnetic field is shown. With increasing the external magnetic field, electronic heat capacity reduces until gμ_B_B = 1.2 eV, and then for the external magnetic field gμ_B_B > 1.2 eV increases. The electronic heat capacity of the buckled silicene monolayer in Fig. 3d displays that for the external magnetic fields of 0.0 eV < gμ_B_B < 0.5 eV and 2 eV < gμ_B_B/t < 2.5 eV the electronic heat capacity decreases and for the external magnetic fields of 0.5 eV < gμ_B_B < 2 eV increases. At a constant external magnetic field at all curves in Fig. 3b,d the electronic heat capacity rises with growing temperature. The results show that the electronic heat capacity of the buckled silicene monolayer is more periodic which is because of more Van Hove singularity in DOS that has been shown in Fig. 2.

**Figure 4:**
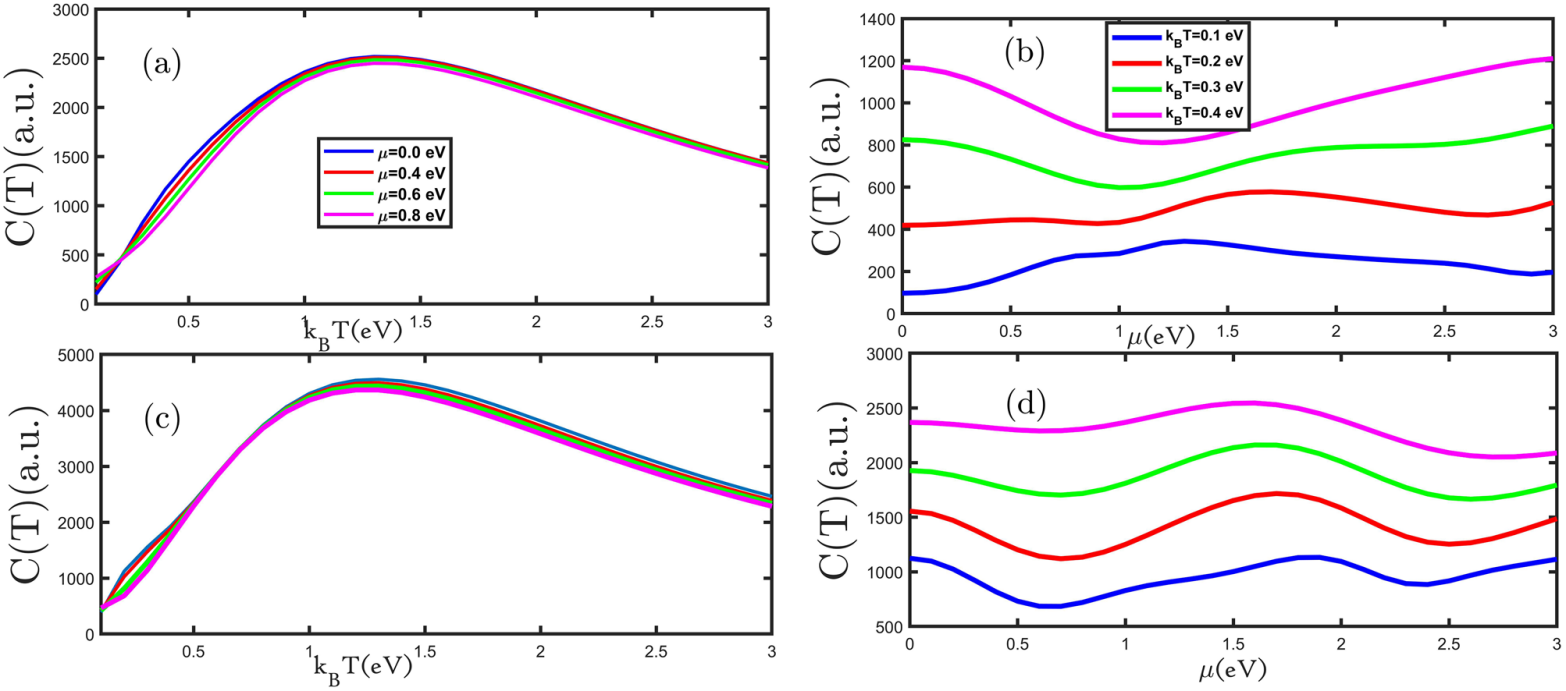
(**a**–**d**) the electronic heat capacity as a function of the temperature (electron doping) for the planar and buckled silicene monolayer in the presence of different electron doping (temperatures), respectively.

In this step, the effect of electron doping on the electronic heat capacity was studied. We study the electronic heat capacity of the planar and buckled silicene monolayer as a function of the temperature for various values of electron doping in Fig. 4a,c. The number of electrons moved from the valance band to the conduction band by increasing temperature, leads to the electronic heat capacity increasing. With more increasing temperature (when the thermal energy has much more than the different energy between the valance band and conduction band) population of electrons in the conduction band increase, and increased the scattering rate of electrons causes a decrease in the electronic heat capacity. In the other words, to understand the situation in which heat capacity increases up to the Schottky anomaly, we need the entropy concept. The entropy of the system helps to claim that at a fairly low temperature, the entropy of the system comprising electrons is very low, while it increases as the temperature is increased up to the Schottky anomaly. At this anomaly, we deal with almost filled states, so heat capacity decreases with temperature. The transition probability of electrons from the lower bands to the upper bands decreases at high temperatures.

At the range of low temperature, in Fig. 4a,c by increasing electron doping, the electronic heat capacity of the planar and buckled silicene monolayer increases. But at high temperatures, due to the predominance of thermal fluctuations and insignificant quantum effects, with the increase of electron doping, the increased scattering rate of electrons decreases as a result of the electronic heat capacity. We have investigated the electronic heat capacity of planar and buckled silicene monolayer as a function of electron doing in Fig. 4b,d. In Fig. 4b the electronic heat capacity of planar silicene monolayer with increasing electron doping is reduced until 0.0 eV < μ < 1.0 eV and then for μ > 1.0 eV grows. Figure 4d shows that the electronic heat capacity for 0.0 eV < μ < 0.75 eV and 2 eV < μ < 2.5 eV is decreased and for 0.75 eV < μ < 2 eV is increased. The sign of doping affects the scattering process, such that positive doping binds up the hole while negative doping attracts electrons. Therefore, we increase the electron (hole) doping, it increases (decreases) the scattering, hence the electronic heat capacity decreases (increases). For Fig. 4b,d in the fixed amount of electron doping, with increasing temperature, electronic heat capacity is growing.

**Figure 5:**
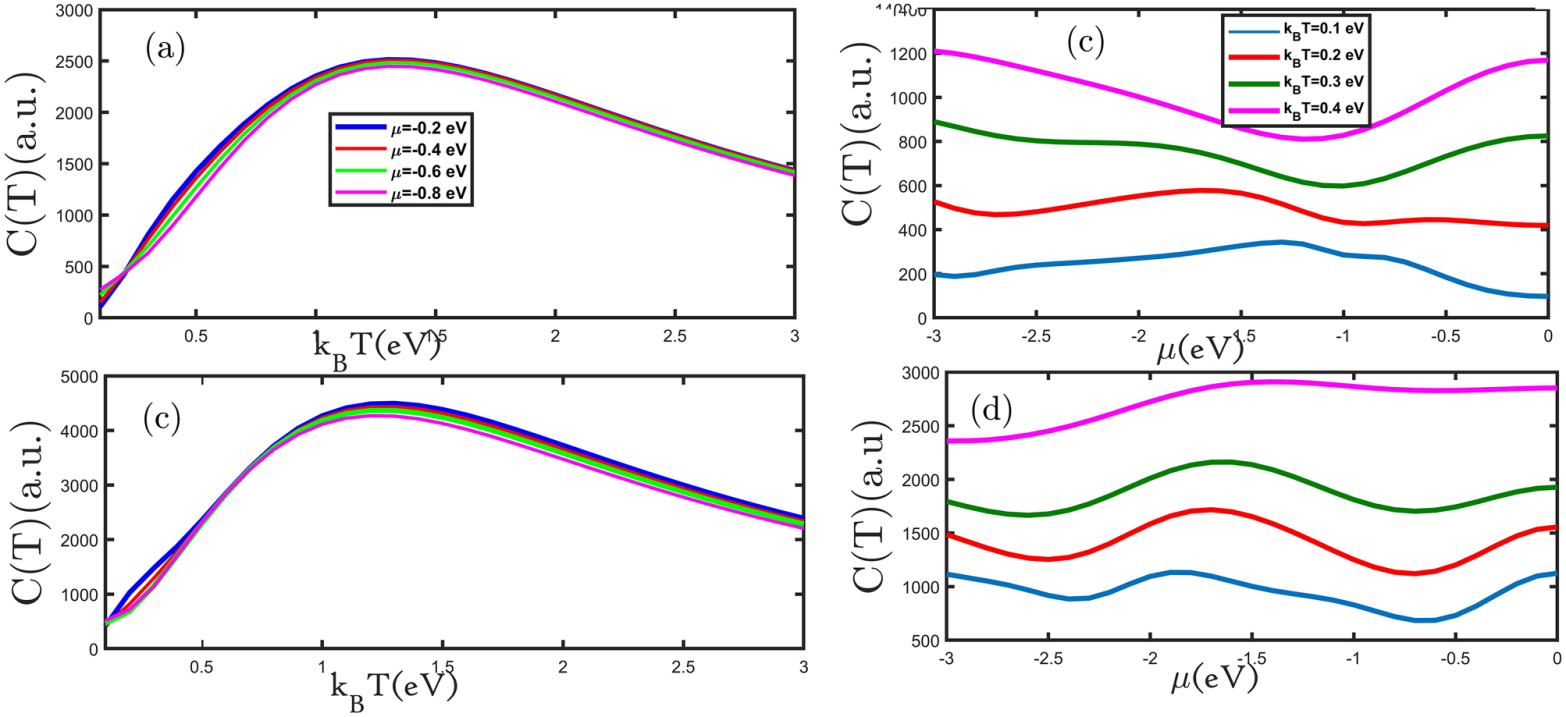
(**a**–**d**) the electronic heat capacity as a function of the temperature (hole doping) for the planar and buckled silicene monolayer in the presence of different hole doping (temperatures), respectively.

We also have examined the temperature dependence of the electronic heat capacity of the planar and buckled silicene monolayer for various values of hole doping in Fig. 5a,c. In Fig. 5a,c, we can see that in all curves Schottky anomaly appears, and by increasing hole doping the Schottky anomaly height is reduced. At a small temperature range, increasing hole doping led to electronic heat capacity being raised. Based on Einstein's model $C(T)\approx {e}^{-{\varepsilon }_{g}/{k}_{B}T}$^33^, we find out that for planar and buckled silicene monolayers, an increase in hole doping is the cause of the increase in metallic properties. To illustrate how the electronic heat capacity changes with hole doping, we plotted the electronic heat capacity of the planar and buckled silicene monolayer in terms of hole doping in Fig. 5b,d. The electronic heat capacity of the planar silicene monolayer for the range of − 3.0 eV < μ < − 1.0 eV is reduced and the range of − 1.0 eV < μ < 0.0 eV is increased (Fig. 5b). For buckled silicene monolayer in Fig. 5d, the electronic heat capacity at the range of − 3.0 eV < μ < − 2.75 eV and − 1.0 eV < μ < − 1.5 eV is reduced and for − 2.75 eV < μ < − 1.0 eV and − 1.5 eV < μ < 0.0 eV is grow. In Fig. 5b,d, at a constant value of hole doping with increasing temperature electronic heat capacity raises.

**Figure 6:**
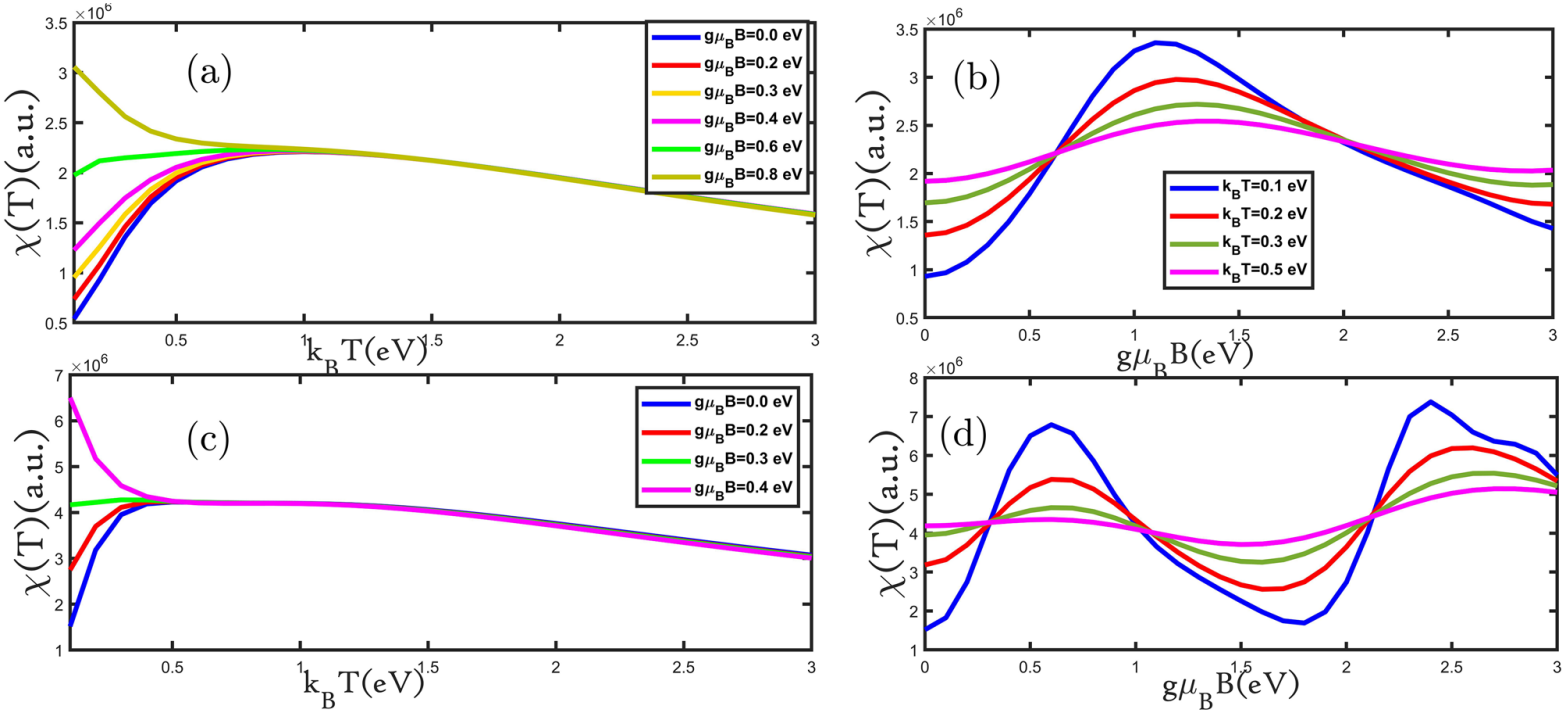
(**a**–**d**) the magnetic susceptibility as a function of the temperature (external magnetic field) for planar and buckled silicene monolayers in the presence of different external magnetic fields (temperatures), respectively.

Magnetic properties of materials are categorized based on their magnetic susceptibility. Susceptibility indicates a substance's ability to become magnetized in response to an external magnetic field. Magnetic susceptibility is determined by χ and is dimensionless. Magnetic materials are classified according to their magnetic susceptibility characteristics: diamagnetic substances which have negative susceptibilities (χ < 0); paramagnetic, superparamagnetic, and ferromagnetic substances which have positive susceptibilities (χ > 0)^30^. Therefore, given the importance of magnetic susceptibility, Fig. 6a,c depict magnetic susceptibility as a function of the temperature for various values of the external magnetic field for the silicene monolayer. The magnetic susceptibility changes with temperature following the Brillouin equation^31^, at a low-temperature range, according to the Brillouin equation magnetic susceptibility is increased with the increasing temperature where electrons behave like quantum particles. At high temperatures, the magnetic susceptibility changes with temperature following the Curie–Weiss law^31^ that the magnetic susceptibility reduces as a function of inverse temperature. In the range of low temperatures, at a constant temperature, increasing the external magnetic field led to magnetic susceptibility raises (Fig. 6a,c). We see obviously at high temperatures in Fig. 6a,b, at a fixed temperature, with the increasing external magnetic field, the magnetic susceptibility is reduced which is similar to the magnetic susceptibility behavior of armchair graphene nanoribbon in the presence of an external magnetic field that reported by Rezania et al.^32^. As we can see, the planar and buckled silicene monolayer is an antiferromagnetic material that has changed to the ferromagnetic phase by applying an external magnetic field. The temperature at which magnetic susceptibility reaches its maximum value is called the Neel temperature. With the phase change of the material to ferromagnetic, the Neel temperature changes to the Curie temperature^27^. We present the magnetic susceptibility dependence of external magnetic field for planar and buckled silicene monolayer in Fig. 6b,d. The magnetic susceptibility of the planar silicene monolayer at the external magnetic field in the value range of 0.0 eV < gμ_B_B < 1.3 eV is grown and at 1.3 eV < gμ_B_B < 3.0 eV is decreased in Fig. 6b. At the value external magnetic field of 0.0 eV < gμ_B_B < 0.5 eV and 2.0 eV < gμ_B_ B < 3 eV with increasing temperature, the magnetic susceptibility is boosted and at the external magnetic field of 0.5 eV < gμ_B_B < 2.0 eV with raising temperature magnetic susceptibility is reduced. The magnetic susceptibility of buckled silicene monolayer at external magnetic fields of 0.0 eV < gμ_B_B < 0.5 eV and 2 eV < gμ_B_B < 2.5 eV is increased and for 0.5 eV < gμ_B_B < 2 eV and 2.5 eV < gμ_B_B < 3.0 eV is decreased (Fig. 6d). In the fixed amount of external magnetic field at 0.0 eV < gμ_B_B < 0.5 eV and 1.0 eV < gμ_B_B < 2.0 eV with increasing temperature the magnetic susceptibility is grown and then at an external magnetic field of 0.5 eV < gμ_B_B < 1.0 eV and 2.0 eV < gμ_B_B < 3.0 eV by increasing temperature the magnetic susceptibility is decreased.

**Figure 7:**
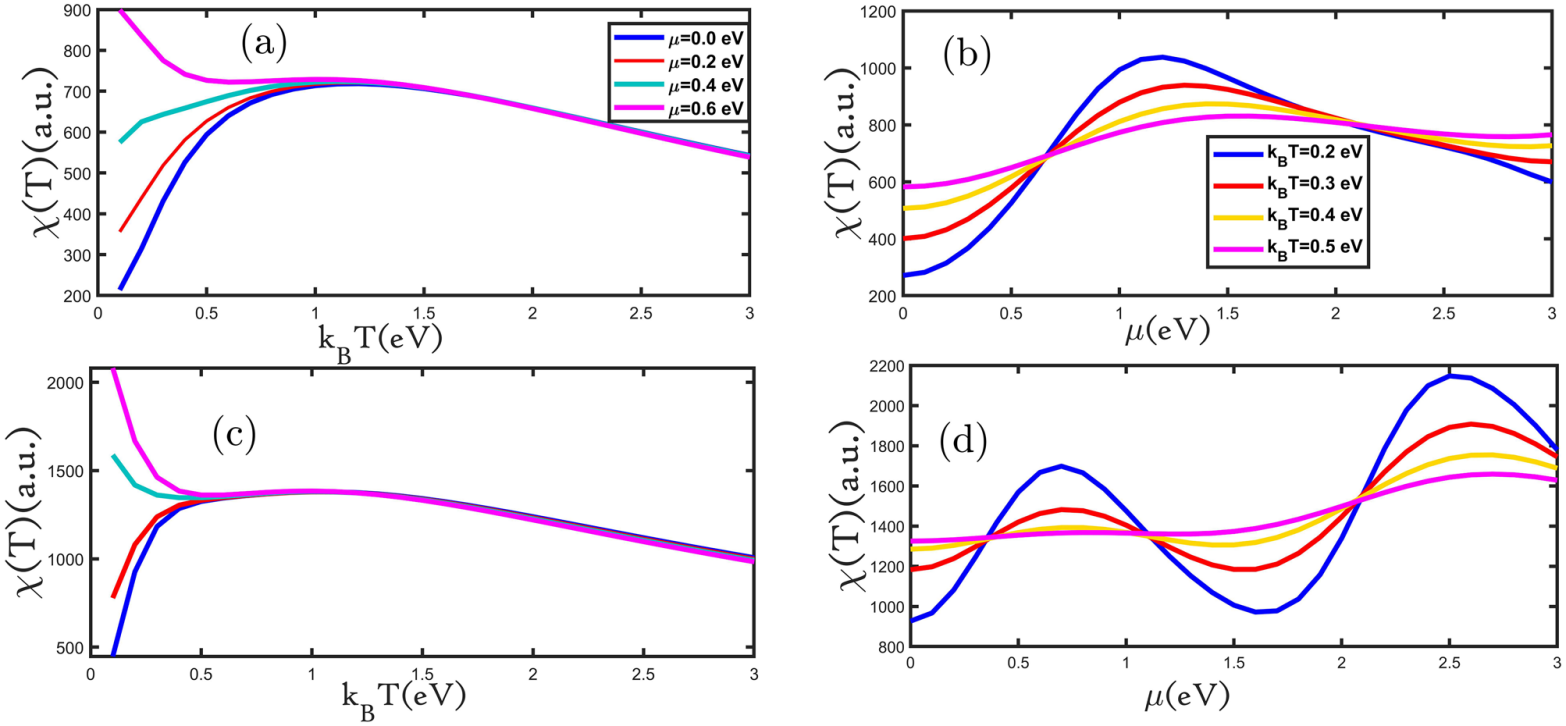
(**a**–**d**) the magnetic susceptibility as a function of the temperature (electron doping) for planar and buckled silicene monolayers in the presence of different electron doping (temperatures), respectively.

In Fig. 7a,c the magnetic susceptibility of planar and buckled silicene monolayer in terms of temperature for different values of electron doping is depicted. In Fig. 7a,c the intensity of the peak that appears in each curve increased with rising electron doping. In the low temperatures range, at a constant temperature, by growing electron doping, the magnetic susceptibility is raised. Also, in the high temperatures range, at a constant temperature with increasing electron doping, the magnetic susceptibility is reduced (in Fig. 7a,c). We examined the magnetic susceptibility of the planar and buckled silicene monolayer as a function of electron doping for different amounts temperatures (Fig. 7b,d). The magnetic susceptibility of the planar silicene monolayer with increasing electron doping is increased at a range of 0.0 eV < μ < 1.0 eV, but for the μ value of 1.0 eV < μ < 3.0 eV it's decreased (in Fig. 7b). In the electron doping range of 0.0 eV < μ < 0.5 eV and 2.0 eV < μ < 3.0 eV with growing temperature, the magnetic susceptibility is increased and for the value of 0.5 eV < μ < 2 eV with increasing temperature, the magnetic susceptibility has reduced. The magnetic susceptibility of the buckled silicene monolayer at 0.0 eV < μ < 0.75 eV and 1.75 eV < μ < 2.5 eV has increased, but for the value of 0.75 eV < μ < 1.75 eV and 2.5 eV < μ < 3 eV has decreased (in Fig. 7d). In the electron doping range of 0.0 eV < μ < 0.5 eV and 1.0 eV < μ < 2.0 eV with growing temperature, the magnetic susceptibility is increased and at 0.5 eV < μ < 1 eV and 2.0 eV < μ < 3.0 eV it is reduced.

**Figure 8:**
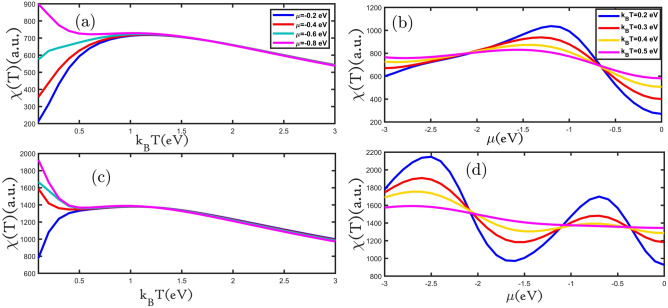
(**a**–**d**) the magnetic susceptibility as a function of the temperature (hole doping) for planar and buckled silicene monolayers in the presence of different hole doping (temperatures), respectively.

Finally, we investigate the effect of hole doping on the magnetic susceptibility of the planar and buckled silicene monolayer. In Fig. 8a,c the magnetic susceptibility of the planar and buckled silicene monolayer is depicted as a function of temperature for different values of hole doping. In Fig. 8a,c we can see that at low temperatures, the magnetic susceptibility increases with temperature according to the Brillouin equation and then decreases with the reverse temperature at higher temperatures according to Curie–Weiss law. The dependence of the magnetic susceptibility on temperature can be divided into two ranges. At a temperature value of 0.0 eV < k_B_T < 0.75 eV, the magnetic susceptibilities with growing hole doping increase, and after its peak with rising hole doping, the magnetic susceptibilities decrease. Resulting of the magnetic susceptibility of the planar and buckled silicene monolayer as a function of hole doping for different temperatures is shown in Fig. 8b,d. The magnetic susceptibility of the planar silicene monolayer increases for the hole doping range of − 3.0 eV < μ < − 1.0 eV and − 1.0 eV < μ < 0.0 eV decreases (in Fig. 8b). At the hole doping range of − 3.0 eV < μ < − 2.0 eV and − 0.75 eV < μ < 0.0 eV, the magnetic susceptibilities increases with increasing temperature, and for − 2.0 eV < μ < − 0.75 eV it's reduced. Figure 8d shows that the magnetic susceptibility of the buckled silicene monolayer increased for hole doping in the range of − 3.0 eV < μ < − 2.5 eV and − 1.5 eV < μ < − 0.75 eV, but for the range of − 2.5 eV < μ < − 1.5 eV and − 0.75 eV < μ < 0.0 eV its decreased. At constant hole doping, at the range of − 2.0 eV < μ < − 1.0 eV and − 0.5 eV < μ < 0.0 eV, the magnetic susceptibilities with growing temperature raised, and for the range of − 3.0 eV < μ < − 2.0 eV and − 1.0 eV < μ < − 0.5 eV with increasing temperature it's reduced.

The type of the majority of charge carriers of the type of carriers n-typed or p-type doped has a significant effect on the thermodynamic properties. Our results show that the type of carriers is changing in different external magnetic fields and doping ranges^33^. Therefore, these changes in the majority of charge carriers cause a change in the order of the dipoles, which makes the process of changing the magnetic susceptibility in terms of external magnetic field and doping not regular.

## Conclusions

To summarize, we computed the electronic and thermodynamic properties of the planar and buckled silicene monolayer by employing the TB and Green’s function method. The temperature, external magnetic field, and electron and hole doping effects on the electronic heat capacity, magnetic susceptibility, and density of states have been examined**.** Our results show that the planer and buckled silicene monolayer have a zero band gap. Also, the electronic heat capacity and magnetic susceptibility change by applying an external magnetic field, and electron and hole doping. Controlling these properties leads to the application of silicene in electronic and spintronic devices.

## Supplementary Information

Supplementary Information.

## Data Availability

We did not use any information and the supporting file includes proof of relationships.
