# Supplementary material for: Effect of external magnetic field and doping on electronic and thermodynamic properties of planer and buckled silicene monolayer
Source: Sci Rep. 2022 Dec 24;12:22277. doi: 10.1038/s41598-022-26353-1 (PMC9789949; doi:10.1038/s41598-022-26353-1)
Supplement: Supplementary file 1 — Supplementary Information. [file 41598_2022_26353_MOESM1_ESM.docx]

1. **Appendix A:**

In the appendix, we attained the TB Hamiltonian matrix Eq.3.for monolayer planar and buckled silicene in the nearest neighbor Approximation. Due to the Eq.1. and Eq.3.the matrix Hamiltonian $H_{AA}^{\sigma}$ and $H_{BB}^{\sigma}$as follows:

(A-1)

$H_{AA}^{\sigma}$=$H_{BB}^{\sigma}$

For monolayer planar and buckled silicene matrix Hamiltonian is given by:

(A-2)

$$H_{s_{A},s_{B}}^{\sigma}=t_{ss\sigma}\left( e^{ik.\Delta_{1}}+e^{ik.\Delta_{2}}+e^{ik.\Delta_{3}} \right)$$

(A-3)

$$H_{s_{A},p_{x,B}}^{\sigma}=-H_{p_{x,A},s_{B}}^{\sigma}=t_{sp\sigma}\left( \sin\varphi e^{ik.\Delta_{1}}+\frac{\sin\varphi}{2}e^{ik.\Delta_{2}}-\frac{\sin\varphi}{2}e^{ik.\Delta_{2}} \right)$$

(A-4)

$$H_{s_{A},p_{y,B}}^{\sigma}=-H_{p_{y,A},s_{B}}^{\sigma}=t_{sp\sigma}\left( \frac{\sqrt{3}}{2}\sin\varphi e^{ik.\Delta_{2}}-\frac{\sqrt{3}}{2}\sin\varphi e^{ik.\Delta_{3}} \right)$$

(A-5)

$$H_{s_{A},p_{z,B}}^{\sigma}=-H_{p_{z,A},s_{B}}^{\sigma}=t_{sp\sigma}\left( \cos\varphi e^{ik.\Delta_{1}}+\cos\varphi e^{ik.\Delta_{2}}+\cos\varphi e^{ik.\Delta_{3}} \right)$$

(A-6)

$$H_{p_{x,A},p_{x,B}}^{\sigma}=\left( t_{pp\sigma}({\sin\varphi)}^{2}-t_{pp\pi}\left( 1-{(sin \varphi)}^{2} \right) \right)e^{ik.\Delta_{1}}+\left( t_{pp\sigma}\frac{({\sin\varphi)}^{2}}{4}-t_{pp\pi}\left( 1-\frac{{(sin \varphi)}^{2}}{4} \right) \right)e^{ik.\Delta_{2}}+\left( t_{pp\sigma}\frac{({\sin\varphi)}^{2}}{4}-t_{pp\pi}\left( 1-\frac{{(sin \varphi)}^{2}}{4} \right) \right)e^{ik.\Delta_{3}}$$

(A-7)

$$H_{p_{y,A},p_{y,B}}^{\sigma}=\left( -t_{pp\pi} \right)e^{ik.\Delta_{1}}+\left( t_{pp\sigma}\frac{3({\sin\varphi)}^{2}}{4}-t_{pp\pi}\left( 1-\frac{3{(sin \varphi)}^{2}}{4} \right) \right)e^{ik.\Delta_{2}}+\left( t_{pp\sigma}\frac{3({\sin\varphi)}^{2}}{4}-t_{pp\pi}\left( 1-\frac{3{(sin \varphi)}^{2}}{4} \right) \right)e^{ik.\Delta_{3}}$$

(A-8)

$$H_{p_{z,A},p_{z,B}}^{\sigma}=\left( t_{pp\sigma}{(\cos\varphi)}^{2}-t_{pp\pi}\left( 1-{(\cos\varphi)}^{2} \right) \right)e^{ik.\Delta_{1}}+\left( t_{pp\sigma}{(\cos\varphi)}^{2}-t_{pp\pi}\left( 1-{(\cos\varphi)}^{2} \right) \right)e^{ik.\Delta_{2}}+\left( t_{pp\sigma}{(\cos\varphi)}^{2}-t_{pp\pi}\left( 1-{(\cos\varphi)}^{2} \right) \right)e^{ik.\Delta_{3}}$$

(A-9)

$$H_{p_{x,A},p_{y,B}}^{\sigma}=\left( -\frac{\sqrt{3}}{4}{(sin \varphi)}^{2} \right)\left( t_{pp\sigma}-t_{pp\pi} \right)e^{ik.\Delta_{2}}+\left( \frac{\sqrt{3}}{4}{(sin \varphi)}^{2} \right)\left( t_{pp\sigma}-t_{pp\pi} \right)e^{ik.\Delta_{3}}$$

(A-10)

$$H_{p_{x,A},p_{z,B}}^{\sigma}=H_{p_{z,A},p_{x,B}}^{\sigma}=\sin\varphi\cos\varphi\left( t_{pp\sigma}-t_{pp\pi} \right)e^{ik.\Delta_{1}}-\frac{\sin\varphi}{2}\cos\varphi\left( t_{pp\sigma}-t_{pp\pi} \right)e^{ik.\Delta_{2}}-\frac{\sin\varphi}{2}\cos\varphi\left( t_{pp\sigma}-t_{pp\pi} \right)e^{ik.\Delta_{3}}$$

(A-11)

$$H_{p_{y,A},p_{z,B}}^{\sigma}=H_{p_{z,A},p_{y,B}}^{\sigma}=\frac{\sqrt{3}}{2}\sin\varphi\cos\varphi\left( t_{pp\sigma}-t_{pp\pi} \right)e^{ik.\Delta_{2}}-\frac{\sqrt{3}}{2}\sin\varphi\cos\varphi\left( t_{pp\sigma}-t_{pp\pi} \right)e^{ik.\Delta_{3}}$$

For monolayer planar silicene $\varphi=90^{\circ}$and monolayer buckled silicene $\varphi=101.7^{\circ}$, we calcalueted TB Hamiltonaian of monolayer silicene of Eq.(A-2) to Eq.(A-11).
